# Supplementary material for: Missing Shapiro steps in topologically trivial Josephson junction on InAs quantum well
Source: Nat Commun. 2021 Jan 4;12:78. doi: 10.1038/s41467-020-20382-y (PMC7782802; doi:10.1038/s41467-020-20382-y)
Supplement: Supplementary file 1 — Supplementary Information [file 41467_2020_20382_MOESM1_ESM.pdf]

Supplementary Information: Missing Shapiro steps in topologically trivial Josephson Junction on InAs quantum well

Matthieu C. Dartiailh[1], Joseph J. Cuozzo[2], William Mayer[1], Joseph Yuan[1],
Kaushini S. Wickramasinghe[1], Enrico Rossi[2], and Javad Shabani[1]
[1] *Center for Quantum Phenomena, Department of Physics, New York University, NY 10003, USA*
[2] *Department of Physics, William & Mary, Williamsburg, VA 23187, USA*

SUPPLEMENTARY FIGURES

Shapiro steps at lower frequency

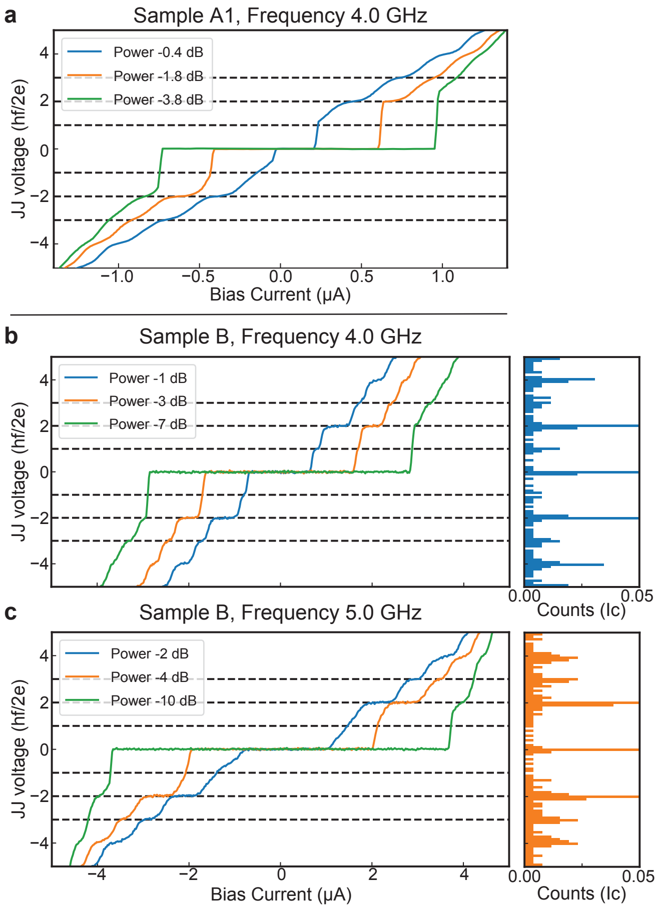

Supplementary Figure 1: **VI characteristic at lower frequency**. **a**, Sample A at 4 GHz, **b, c**, Sample B at 4 and 5 GHz and corresponding histogram of the voltage (the color match the VI legend).

Supplementary Fig. 1 presents data on both device A1 and B at the lowest frequency achievable in our setup. This limit is mostly due to the weak coupling of the microwave antenna to the device at low frequency.

On both devices, one can observe the same missing first step described in the main text. In addition, on device B the third step appears partially suppressed as illustrated in the histograms. At 4 GHz, the suppression appears nearly total, while at 5 GHz the third step is slightly weaker than the fourth one when it is expected to be stronger. This is consistent with data presented at 6 GHz in the main text and allows us to rule out possible experimental setup imperfections to explain the partial suppression of the third step.

Additional data on device A2.

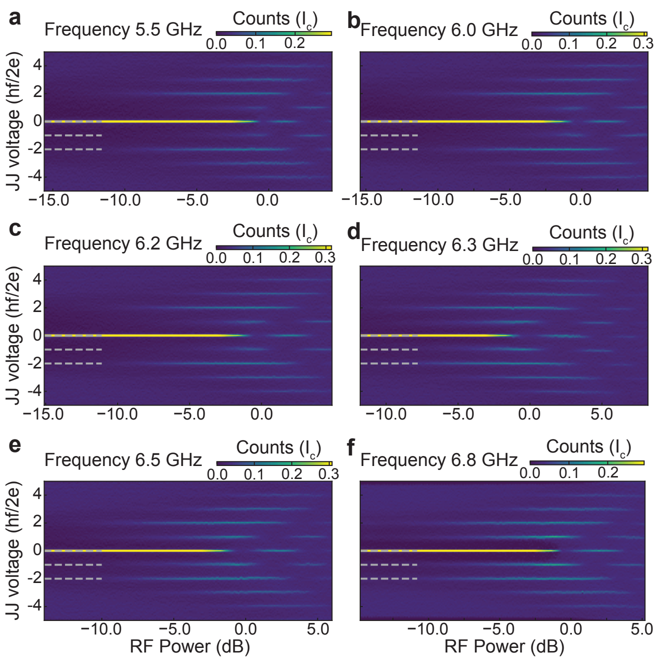

Supplementary Figure 2: **Frequency cross-over in sample A2. a-f** Histograms of the voltage as a function of the microwave power for device A2 at 650 mK for 6 different frequencies illustrating the recovery of the first step as frequency is increased.

In Supplementary Fig. 2, we present additional data on device A2 at 650 mK. As the frequency of the microwave drive is increased the first step is recovered at lower and lower power allowing us to pinpoint the cross-over frequency around 6.5 GHz, which yields a maximal amount of 4π current of 35 nA.

Sub-harmonic steps

A JJ with a high transparency is expected to have a forward skewed CPR as can be seen from Equation (4) in the main text. The CPR can be directly measured by embedding the JJ in a superconducting interference

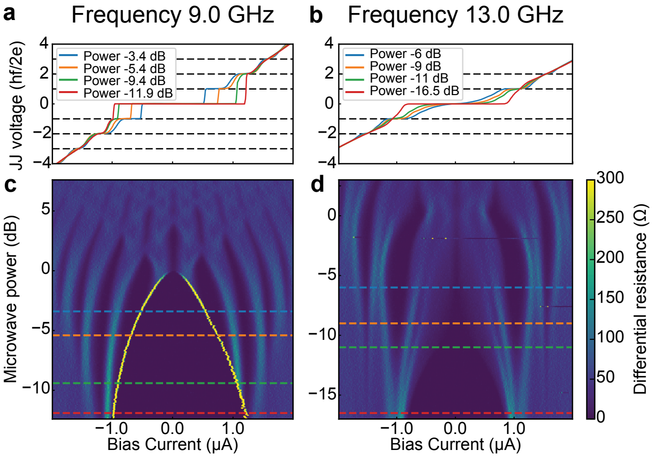

Supplementary Figure 3: **Fractional step observation. a, b** Voltage-current characteristics in the presence of a microwave radiation of device A1 and **c, d** differential resistance map obtained by numerical differentiation as a function of the current bias and applied microwave power. At high frequency, **b, d**, weak half Shapiro steps appear in the VI characteristic which are visible in the differential resistance map.

device (SQUID), whose second JJ has a much larger critical current. Such measurements have been carried out on graphene-based JJ [1], TI based JJ [2, 3], InAs nanowire [4] and in our devices [5].

The anharmonicity associated with forward skewness of the CPR has been predicted to lead to the appearance of subharmonics Shapiro steps at high frequency drives [6]. Such subharmonic steps have been observed in several systems [7–10], in which they were associated with a skewed CPR. In Supplementary Fig. 3, we present the VI characteristic and differential resistance as a function of a.c. power and bias current of device A1 at 9 GHz and 13 GHz. While at 9 GHz, only integer steps are visible, at 13 GHz a weak half-step is visible in the VI. The existence of the subharmonic step is also visible in the differential resistance map as a splitting of the resistance peak separating two integer steps. This signature provides an additional experimental signature of the high transparency of our junctions.

RSJ Model

The dynamics of a current-driven JJ can be treated within a semiclassical description where the current is carried in three parallel channels: a resistive channel describing dissipative current, a capacitive channel describing charge accumulation on the superconducting leads across the weak link, and a supercurrent channel describing current mediated by Cooper pairs. Our JJ has a small geometric capacitance ($C \sim 1$ fF) corresponding to the overdamped regime; thus, we neglect the capacitive channel. Driving our JJ with an ac current, we can use Kirchoff's junction law to write

$$I_{\text{drive}}(t) = I_R(\dot{\phi}) + I_s(\phi) \tag{1}$$

where ϕ is the phase difference between the two superconducting electrodes, $I_{\text{drive}}(t) = I_{\text{dc}} + I_{\text{ac}}\sin(2\pi f_{\text{ac}} t)$ is the external driving current, $I_{\text{R}}(\dot{\phi}) = \frac{\hbar}{2eR}\dot{\phi}$ is the current in the resistive channel, and $I_s(\phi)$ is the supercurrent contribution. Then the equation governing the JJ's dynamics is

$$\frac{\hbar}{2eI_cR}\dot{\phi} = \hat{I}_{\text{dc}} + \hat{I}_{\text{ac}}\sin(2\pi f_{\text{ac}} t) - \hat{I}_s(\phi), \tag{2}$$

where the hats denote currents normalized by the critical supercurrent I_c.

Justification of the two channels model

As we discuss in the main text the two channels model that we use to describe the dynamics of the JJs is justified by the nature of the spectrum of our junctions, that have a width W much larger than L and than the bulk coherence length ξ. Supplementary Fig. 4 shows the spectrum of the ABS states for a junction with $L = 100$ nm and $W = 500$ nm. We see that there are 2-4 modes that are well separated by the quasicontinuous spectrum at $E_n \approx \Delta$. These are the modes for which $\xi_{n_y} > L$. We also see that these modes are the ones with the largest transparency i.e. the smallest gap for $\phi = \pi$, and therefore the ones with the largest probability to undergo a LZT at $\phi = \pi$. The combination of high transparency and distance from the quasicontinuum, is what allows to provide a robust 4π contribution to the Josephson current at low powers and frequencies.

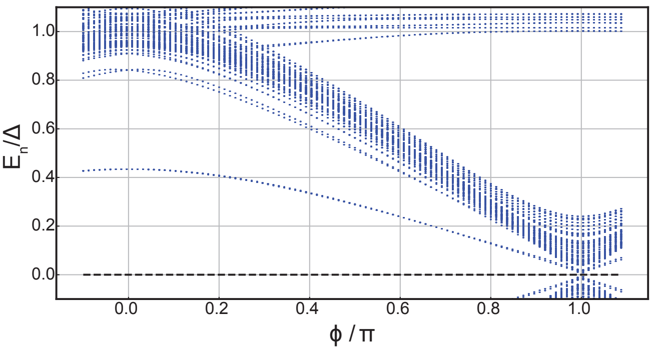

Supplementary Figure 4: **ABS spectrum for a wide JJ.** The calculation was done for W = 500 nm, L = 100 nm, Δ = 0.3 meV, λ_{SOC} = 7.5meV nm. Here we used $m = 0.04 m_e$ and doping $n = 8 \times 10^{11}$ cm^{-2}.

Two channels RSJ model

In JJ's, the supercurrent is mediated by Andreev bound states (ABS) with energy given by

$$E_{\text{ABS}} = \pm\Delta\sqrt{1 - \tau\sin^2(\phi/2)} \tag{3}$$

where Δ is the SC gap and τ is the transparency. The spectrum according to Equation (3) exhibits a gap $2\Delta\sqrt{1-\tau}$ at $\phi = \pi$. The supercurrent carried by a single ABS at zero temperature is given by a skewed sinusoidal CPR,

$$I_{\text{ABS}} = \frac{e\Delta}{2\hbar} \frac{\tau \sin(\phi)}{\sqrt{1-\tau \sin^2(\phi/2)}}. \quad (4)$$

In what follows, we assume the distribution of mode transparencies to be bimodal, where the two lobes of the bimodal distribution curve are centered around a low transparency τ_{low} and a high transparency $\tau_{\text{high}} > \tau_{\text{low}}$. We further assume the majority of the modes reside near τ_{low}. Now, we simplify the contribution made to the supercurrent channel by letting

$$I_{\text{low}\,\tau} = \frac{e\Delta}{2\hbar} \sum_{\tau \in \mathcal{T}_{\text{low}}} \frac{\partial E_\tau}{\partial \phi} \approx n I_{2\pi} \sin(\phi)$$

and

$$I_{\text{high}\,\tau} = \frac{e\Delta}{2\hbar} \sum_{\tau \in \mathcal{T}_{\text{high}}} \frac{\partial E_\tau}{\partial \phi} \approx \frac{(1-n) I_{\tau_{\text{high}}} \sin(\phi)}{\sqrt{1-\tau_{\text{high}} \sin^2(\phi/2)}}$$

where $\mathcal{T}_{\text{low}}$ and $\mathcal{T}_{\text{high}}$ are the set of transparencies of modes belonging to the lower τ and higher τ modes, respectively and n is the fraction of critical current contributed by the effective low-transparency mode and I_c is the critical current. Thus, we consider two *effective* modes: a low-transparency mode with a sinusoidal CPR and a high-transparency mode with a skewed CPR determined by an *effective* transparency $\tau = \tau_{\text{high}}$. Then we write our supercurrent as:

$$I_s = I_c \left(\frac{n}{\alpha_0} \sin(\phi) + \frac{1-n}{\alpha_\tau} \frac{\sin(\phi)}{\sqrt{1-\tau \sin^2(\phi/2)}} \right), \quad (5)$$

We have included normalizations α_0 and α_τ that are determined by,

$$\alpha_0 = \frac{1}{\sin(\tilde{\phi}_{\text{max}})}, \quad \alpha_\tau = \frac{\sqrt{1-\tau \sin^2(\tilde{\phi}_{\text{max}})}}{\sin(\tilde{\phi}_{\text{max}})} \quad (6)$$

where $\tilde{\phi}_{max}$ is such that

$$\max \left(n \sin(\phi) + (1-n) \frac{\sin(\phi)}{\sqrt{1-\tau \sin^2(\phi/2)}} \right)$$
$$= n \sin(\tilde{\phi}_{\text{max}}) + (1-n) \frac{\sin(\tilde{\phi}_{\text{max}})}{\sqrt{1-\tau \sin^2(\tilde{\phi}_{\text{max}}/2)}}$$

These normalizations remove the inherent difference between the sinusoidal and skewed CPR's contribution to the critical current, allowing n to accurately determine the fraction of critical current coming from the low and high transparency channels.

Landau-Zener Processes

In a two-level quantum system, Landau-Zener processes describe diabatic energy level transitions. Generally, the Landau-Zener transition (LZT) probability will depend on the difference in energy between the two states and the rate at which the dynamical variable changes: a small energy gap and rapid evolution of the dynamical variable are favorable conditions for an LZT to occur. We can treat the ground state and excited state of a single ABS with transparency τ as a two-level quantum system and solve for the LZT probability at the avoided crossing [11]:

$$P_{\text{LZT}}(t) = \exp\left(-\pi \frac{\Delta(1-\tau)}{e\,|V(t)|} \right). \quad (7)$$

Here we neglect interference effects due to phase fluctuations and coherence between LZTs.

A successful LZT will change the sign of the supercurrent contribution due to the ABS mode undergoing the transition. For high transparency modes, LZT probability can be significant because of the small gap at the avoided crossing. We model the collective behavior of the high-transparency modes by considering a single *effective* LZT in our calculations occurring at avoided crossings. Thus, we take our supercurrent to be given by,

$$I_s = I_c \left(\frac{n}{\alpha_0} \sin(\phi) + s \frac{(1-n)}{\alpha_\tau} \frac{\sin(\phi)}{\sqrt{1-\tau \sin^2(\phi/2)}} \right), \quad (8)$$

where $s = \pm$ controls the sign flip due to an LZT. We solve Equation (2) dynamically to account for LZTs at the avoided crossing of the effective high-transparency mode.

Additional simulation results

Supplementary Fig. 5 shows the main results from the main text along with histograms of Josephson junction voltage as function of ac power. We observe odd steps gradually suppressed at low driving frequencies and low power which qualitatively agrees with experimental results.

Before discussing the frequency dependence of $V(t_{\text{LZT}})$ in the two modes model, we can start with a simplified picture where we only consider a purely sinusoidal CPR in the absence of LZ transitions (i.e. $n = 1$). Phase and instantaneous voltage across the JJ for $f_J = 2\,e\,I_c\,R_n = 3.1\Delta$ (corresponding to $I_c\,R_n$ for device A) and $f_J = 0.94\Delta$ for driving frequencies $f_{\text{ac}} = 0.2 f_J$ and $0.05 f_J$ are shown in Supplementary Fig. 6. We notice that the peaks of the resonances in V correspond closely

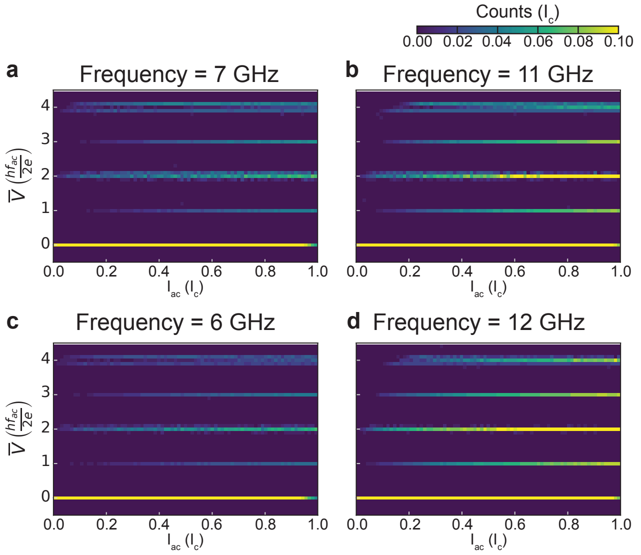

Supplementary Figure 5: **Extended theoretical results** Histogram of Josephson junction voltage as a function of power at a fixed ac driving frequency with device A1 parameters **a,b** with device B parameters **c, d**.

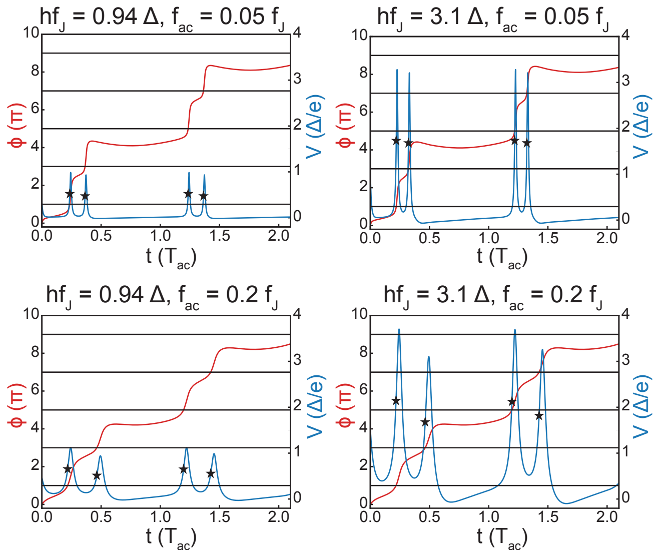

Supplementary Figure 6: **Phase and instantaneous voltage across the JJ as function of time**. We only consider the sinusoidal part of Equation (8) ($n = 1$) for various Josephson frequencies and driving frequencies as indicated in the title of each panel. The black stars indicate the value of V at $\phi = \pi[2\,\pi]$

to hf_J/e and are very weakly dependent on driving frequency. Numerically, we observe that these dependencies survive when we include a skewed CPR and LZTs.

In Supplementary Fig. 7a and b, we consider again our two modes model and show the IV curves for the time-average voltage across the junction (solid lines) and the time-average voltage at the avoided crossing $\overline{V}(t_{\rm LZT})$

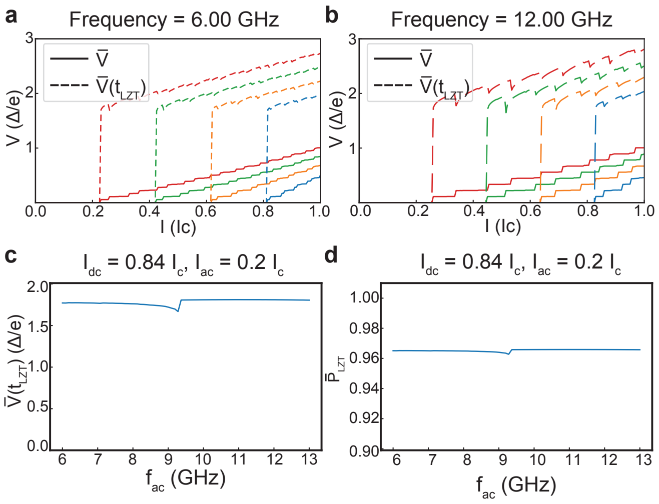

Supplementary Figure 7: **Independence of $V_{\rm LZT}$ on the drive frequency.** Shapiro steps (solid lines) and time-average instantaneous voltage at the avoided crossing $\overline{V}(t_{\rm LZT})$ (dashed lines) for sample B parameters for **a** 6 GHz and **b** 12 GHz. Considered here are the following powers: $I_{\rm ac} = 0.2I_c$ (blue), $I_{\rm ac} = 0.4I_c$ (orange), $I_{\rm ac} = 0.6I_c$ (green), and $I_{\rm ac} = 0.8I_c$ (red). **c** Frequency dependence of $\overline{V}(t_{\rm LZT})$ at $I_{\rm dc} = 0.84I_c$ and $I_{\rm ac} = 0.2I_c$. **d** Frequency dependence of $\overline{P}_{\rm LZT}$ at $I_{\rm dc} = 0.84I_c$ and $I_{\rm ac} = 0.2I_c$.

(dashed lines) where the LZT probability is evaluated. We find that $\overline{V}(t_{\rm LZT})$ has a dependence on the dc current bias and power, but the difference between the two frequencies is the $I_{\rm dc}$ corresponding to the center of a Shapiro step (i.e. the step widths increase with increasing frequency).

To compare LZT probabilities at different frequencies, we consider a fixed dc current bias and power corresponding to the lowest order steps in Supplementary Fig. 7a, b. The kink feature in Supplementary Fig. 7c and d indicates a crossover from the first step at high frequency to the second step at low frequency (where the first step is suppressed). Thus, the LZT process is not suppressed at high frequencies; rather, we can understand the emergence of odd steps at high frequencies by considering our junction having both 2π and 4π periodic channels. For such a junction, there is crossover frequency $f_{4\pi} = 2eR_nI_{4\pi}/h$ (as described in the main text) which determines whether one observes a suppressed first step or not (i.e. if $f_{\rm ac} < f_{4\pi}$ then the first step is suppressed; otherwise, the first step is observed). One can think of this crossover equivalently as $hf_{\rm ac} < 2eR_nI_{4\pi} \equiv \pi\Delta_{4\pi}$. Then the condition for the observation of a suppressed first step is the energy of the photon irradiating the JJ is less than the 4π gap $\Delta_{4\pi}$. In our setup, the high transparency modes have very high Landau-Zener transition probabilities so they are effectively 4π-periodic modes. Thus, we can introduce a similar $\Delta_{4\pi}$ determined by the number of effective 4π

modes in our junction and the argument above follows to describe the frequency dependence of the suppressed first step.

Effect of intermediate LZT's probabilities

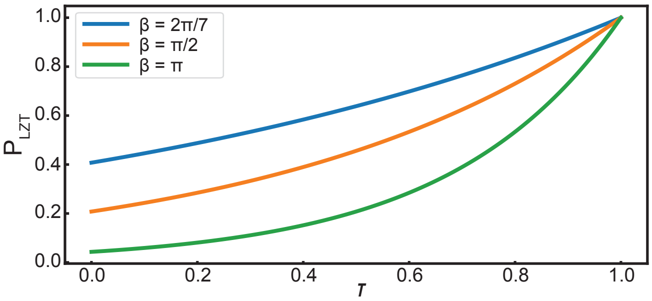

Supplementary Figure 8: **Probability of an LZ transition as a function of the transparency τ.** The result presented are obtained for fixed $\beta = \frac{\pi\Delta}{e|V|}$. The value of β was chosen based on typical values for $|V|$ at $\phi = \pi$ where a transition is allowed in the simulations.

For a simple visualization of the LZT probabilities dependence on transparency, we can ignore the fluctuations in the value of $|\dot{\phi}|$ as ϕ sweeps through odd integer multiples of π, and can consider

$$P_{\rm LZT} = \exp\left(-\beta(1-\tau)\right) \tag{9}$$

where $\frac{2\pi}{7} < \beta < \pi$ is a constant. We have chosen this range of β based on possible values of V giving rise to an LZT in our simulations. Results for various values of β are shown in Supplementary Fig. 8.

One may consider the effect of a transparency that is not near unity i.e. the effect of a LZT probabilty that is not near unity. If we assume $|V(t_{\rm LZT})| = 1.8\Delta/e$ then for transparency < 0.982, the LZT probability takes on values < 0.97 leading to departures from quantized values of induced voltage–integer multiples of $\frac{hf_{\rm ac}}{2e}$ [12]. Supplementary Fig. 9 shows VI curves for $\tau = 0.9$ and $n = 0.95$ using device A1 values for I_c and R_n. Results are similar for device B. Clearly Shapiro steps are not well defined, and even-integer steps generally show a greater deviation from quantized values. The experimental devices have very robust, quantized steps implying only high transparency modes effectively participate in Landau-Zener processes. It is worth noting that this estimate for a lower bound on transparency is rooted in a somewhat arbitrary choice for a lower bound $P_{\rm LZT}$ leading to departures from quantized values of $\overline{V}$ and should not be considered a lower bound for transparencies where missing steps can be observed.

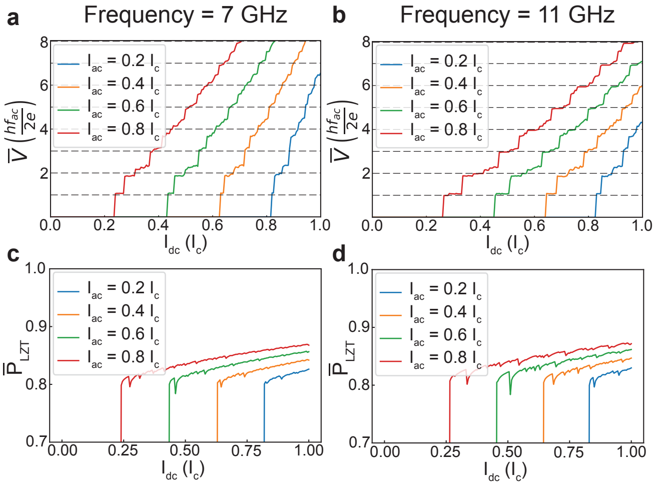

Supplementary Figure 9: **Poor Shapiro steps quantization for intermediate transparencies. a,b** VI characteristics for device A1 using a lower transparency $\tau = 0.9$ and $n = 0.95$. Simulations at lower transparency show deteriorated quantized steps due to a reduced probability of LZTs. **c,d** Time-averaged probability of LZTs as the phase passes an avoided crossing. Clearly, the probability of an LZT must be very near unity to observe sharp quantized steps.

SUPPLEMENTARY REFERENCES

[1] English, C. D. *et al.* Observation of nonsinusoidal current-phase relation in graphene Josephson junctions. *Phys. Rev. B* **94**, 115435 (2016).

[2] Sochnikov, I. *et al.* Nonsinusoidal Current-Phase Relationship in Josephson Junctions from the 3D Topological Insulator HgTe. *Phys. Rev. Lett.* **114**, 066801 (2015).

[3] Kayyalha, M. *et al.* Highly skewed current–phase relation in superconductor–topological insulator–superconductor Josephson junctions. *npj Quantum Mater.* **5**, 1–7 (2020).

[4] Spanton, E. M. *et al.* Current–phase relations of few-mode InAs nanowire Josephson junctions. *Nature Phys* **13**, 1177–1181 (2017).

[5] Mayer, W. *et al.* Gate controlled anomalous phase shift in Al/InAs Josephson junctions. *Nat Commun* **11**, 1–6 (2020).

[6] Askerzade, I. N. Effects of anharmonicity of current-phase relation in Josephson junctions (Review Article). *Low Temperature Physics* **41**, 241–259 (2015).

[7] Wiedenmann, J. *et al.* 4π-periodic Josephson supercurrent in HgTe-based topological Josephson junctions. *Nature Communications* **7** (2016).

[8] Snyder, R. *et al.* Weak-link Josephson Junctions Made from Topological Crystalline Insulators. *Phys. Rev. Lett.* **121**, 097701 (2018).

[9] Lee, G.-H., Kim, S., Jhi, S.-H. & Lee, H.-J. Ultimately short ballistic vertical graphene Josephson junctions. *Nat Commun* **6**, 1–9 (2015).

[10] Panghotra, R. *et al.* Giant fractional Shapiro steps in anisotropic Josephson junction arrays. *Commun Phys* **3**, 1–8 (2020).

[11] Averin, D. & Bardas, A. ac josephson effect in a single quantum channel. *Phys. Rev. Lett.* **75**, 1831–1834 (1995).

[12] Domínguez, F., Hassler, F. & Platero, G. Dynamical detection of Majorana fermions in current-biased nanowires. *Phys. Rev. B* **86**, 140503 (2012).
